# High school drinking mediates the relationship between parental monitoring and college drinking: A longitudinal analysis

**DOI:** 10.1186/1747-597X-3-6

**Published:** 2008-03-07

**Authors:** Amelia M Arria, Vanessa Kuhn, Kimberly M Caldeira, Kevin E O'Grady, Kathryn B Vincent, Eric D Wish

**Affiliations:** 1 Center for Substance Abuse Research (CESAR), University of Maryland, College Park, MD, USA; 2 Department of Health Policy and Management, Johns Hopkins University Bloomberg School of Public Health, Baltimore, MD, USA; 3 Department of Psychology, University of Maryland, College Park, MD, USA

## Abstract

**Background:**

College drinking is a significant public health problem. Although parental monitoring and supervision reduces the risk for alcohol consumption among younger adolescents, few studies have investigated the impact of earlier parental monitoring on later college drinking. This study examined whether parental monitoring indirectly exerts a protective effect on college drinking by reducing high school alcohol consumption.

**Methods:**

A longitudinal cohort of 1,253 male and female students, ages 17 to 19, attending a large, public, mid-Atlantic university was studied at two time points. First, data on high school parental monitoring and alcohol consumption were gathered via questionnaire during the summer prior to college entry. Second, during the first year of college, past-year alcohol consumption was measured via a personal interview. Multiple regression models tested the relationship between parental monitoring and past year alcohol use (i.e., number of drinks per drinking day).

**Results:**

Holding constant demographics, SAT score, and religiosity, parental monitoring had a significant protective effect on both high school and college drinking level. However, the association between parental monitoring and college drinking level became non-significant once high school drinking level was held constant.

**Conclusion:**

While parental monitoring did not directly influence college alcohol consumption, evidence for mediation was observed, whereby parental monitoring had an indirect influence on college drinking through reductions in high school drinking. Initiatives that promote effective parenting might be an important strategy to curb high-risk drinking among older adolescents. More research is needed to understand the nature and degree of parent-child communication that is necessary to extend the protective influence of parents into the college years.

## Background

Underage alcohol consumption has received a significant amount of attention as a major public health concern [1]. High risk drinking in adolescence is associated with a variety of adverse consequences, including motor vehicle injury and death, suicide, sexual assault, high-risk sexual activity, and neurodevelopmental impairment [2-7]. Drinking in early adolescence is also known to increase the risk for alcohol dependence [8,9].

While drinking alcohol in college is often seen as an expected rite of passage, heavy drinking among college students adversely affects students' health, safety, and academic performance [10,11]. The 2005 *Monitoring the Future *survey indicated that while 8.6% of male and 2.3% of female full-time college students drink every day, a much larger proportion have had five or more drinks in a row in the past two weeks (50.1% of males and 34.4% of females) [12]. Unfortunately, the trend line for this indicator of heavy episodic drinking has remained fairly flat and hovered in the region of 40% on average over the last twenty-six years, with a peak of 45.4% in 1984 [13]. As Schulenberg et al. [14] describes, the developmental transition from high school to college and the achievement of milestones beyond college can be compromised by heavy alcohol consumption during the first few years of college. Moreover, recent studies have estimated that one-quarter to one-third of college students meet standard criteria for an alcohol use disorder and few seek treatment while attending college [15-17].

Taken together, the mass of scientific literature regarding college student drinking makes the problem appear almost intractable unless new prevention and intervention strategies are developed and embraced by college administrators, parents, and health care professionals. To this end, researchers have attempted to identify risk factors which then could become targets of interventions. Several cross-sectional studies have identified sociodemographic and other individual-level characteristics that are associated with increased risk for excessive alcohol consumption among college students. For instance, males are heavier drinkers than females [18], White students have higher rates of drinking than minority groups, and members of sororities and fraternities drink significantly more than non-members [19,20]. Consistent with other findings showing a protective effect of religiosity on alcohol consumption among high school students [21], Patock-Peckham et al. [22] found that intrinsic religiosity was associated with a lower risk for college students' drinking.

In addition to sociodemographic characteristics, several studies have shown that high school drinking patterns are highly predictive of college drinking patterns; however, surprisingly few longitudinal studies have been conducted on the risk factors for escalation of drinking during the transition from high school to the first year of college. The *Monitoring the Future *survey, while very useful for describing trends in drinking and identifying differences between college and non-college attending peers [12,19], is limited in scope with respect to possible predictors of college drinking patterns. Baer et al. [23] observed that being male, having Greek system affiliations, and having conduct problems were risk factors for increased drinking in college among a selected sample of 366 high school students who were heavy drinkers. Sher and Rutledge [24] conducted a much larger study of over 3,000 college students using a systematic sampling design, and found a high degree of continuity in the level of alcohol consumption between high school and the first semester of college. Moreover, 54% of the variance in heavy college drinking could be predicted by sex, race, pre-college cigarette use, religiosity, peer drinking norms and ease of obtaining alcohol in high school.

None of the aforementioned longitudinal studies explored whether pre-college parenting practices influence the risk for drinking in college students, despite the substantial body of literature demonstrating the important influences of family characteristics, especially parental drinking behaviors, parent-child relationships, communication, and parental monitoring, on the risk for underage alcohol use [25-29]. Using both cross-sectional and prospective designs, studies have consistently demonstrated that effective parenting practices have a strong impact on reducing the risk of early adolescent alcohol consumption. Different facets of effective parenting have all been shown to be important, including parental monitoring and supervision, expression of unambiguous disapproval of underage drinking, and low levels of parent-child hostility. For example, Nash et al. [30] studied a cohort of high school students (all in 9^th ^grade at the start of the study) and found that higher levels of parental disapproval of alcohol use were associated with lower levels of alcohol use in 12^th ^grade, as compared to students whose parents had low levels of disapproval. The results of that study also suggested that the effects of parental disapproval were mediated by lower levels of peer influence over drinking behaviors [30]. Additionally, Chilcoat and Anthony [31] evaluated 8 to 10 year old children during a three-year period and found that increased levels of parental monitoring and supervision were associated with a 1.6-fold reduction in drug use initiation even holding constant baseline monitoring. In that study, adolescents in the highest parental monitoring quartile experienced a 2-year delay in the onset of drug use compared to adolescents in the lowest quartile of parental monitoring. Similarly, Guo et al. [32] studied a youth cohort from age 10 through 21 and observed that clear rules and close parental monitoring during early adolescence were associated with lower risk for alcohol abuse and dependence during later adolescence. As a final example, Beck et al. [33] found that teens aged 12 to 17 who were monitored more closely were less likely to drink than their less-monitored counterparts, even when controlling for age, gender, drinking at baseline, and being in various high-risk situations, such as seeing teens drink, hanging out with friends who drink, and riding with a driver who had been drinking.

Although the bulk of evidence on parental monitoring and decreased risk for alcohol consumption stems from research on young adolescents, a few studies involving college students warrant mention. First, Turrisi et al. [28] found evidence that teens whose parents who were educated about binge drinking and communication strategies with their teenager experienced fewer alcohol-related problems than a control group. Second, Weitzman et al. [34] analyzed changes in drinking behavior and found that parental attitudes and use were related to a college student's likelihood of engaging in binge drinking. This study, however, did not look at specific parental supervision or parental monitoring behaviors and measures. Third, Sessa [35] conducted a small study of 106 male college students and found that higher perceived parental monitoring during college was related to less alcohol and marijuana use among commuter students, but not residential students. Lastly, in a small convenience sample of college students, Jung [36] observed a correlation between closeness of the parent-student relationship and similarity between parent and student alcohol consumption.

The present study aimed to determine the extent to which the level of parental monitoring and supervision during the last year of high school might account for variation in alcohol consumption during the first year of college. Consistent with prior literature, we expected that students who experienced higher levels of parental monitoring and supervision during their last year in high school would consume less alcohol during high school. Moreover, we hypothesized that pre-college alcohol consumption levels would significantly predict the level of alcohol consumption in college. Lastly, we tested the hypothesis that parental monitoring would exert a significant protective, albeit indirect, effect on college drinking that would be mediated by reductions in high school drinking level.

## Methods

### Participants

Data were gathered as part of the College Life Study, an ongoing longitudinal prospective investigation of college student health risk behaviors, including alcohol and other drug use. A two-stage sampling design was used to ascertain the sample. First, 3,401 incoming first-time, first-year students ages 17 to 19 completed a screening survey during new student orientation in the summer prior to entering a large, public university in the mid-Atlantic region of the United States. The first-stage response rate was 89%. The initial sample represented almost 90% of the incoming class, and did not differ significantly from the class with respect to demographic characteristics [37]. Second, a stratified random sample of screener participants was selected for longitudinal followup, with oversampling for experienced drug users. Sampling weights were calculated so that prevalence estimates could be adjusted for bias introduced by oversampling. The second-stage response rate was 86% of those contacted for follow-up. The follow-up sample (n = 1,253) was interviewed face-to-face by a trained interviewer at some time during their first year of college. Students were paid $5 for completing the screener survey and $50 for completing the personal interview. The study was reviewed and approved by the University Institutional Review Board, a federal Certificate of Confidentiality was acquired, and informed consent was obtained from participants at all stages. Additional details describing the recruitment, sampling methods and representativeness of the sample can be found elsewhere [37].

### Measures

*Precollege background variables *were obtained from the screener survey (race, mother's education level, religiosity) and from university administrative datasets (sex, SAT score) as permitted by informed consent. Race was assessed by the question "How would you describe yourself?" where the respondent was able to choose multiple racial/ethnic categories. Because of the preponderance of Whites in our sample, and the fact that very few students describing themselves as having multi-racial backgrounds, race was dichotomized as White vs. non-White for the present analyses. Mother's education level was used as a proxy for socio-economic status. The combined SAT score was used as a continuous measure representing academic achievement in high school; for ease of interpreting results in the present analyses, SAT score was arithmetically transformed by dividing by 100. Finally, religiosity was measured by a single question "How important is religion in your life?" modeled after the *Monitoring the Future *survey [12]. Response options were "not important," "slightly important," "moderately important," or "extremely important." For ease of interpretation, religiosity was dichotomized as low (not important or slightly important) vs. high (moderately or extremely important).

*Living situation in college *was assessed during the personal interview with the question "Since coming to college, how would you describe your living situation?" Because the presence of parental supervision was salient to the aims of this study, living situation was dichotomized as living with parents or other relatives (*n *= 85) versus living in other situations (*n *= 1,168). The latter group consisted primarily of students living in campus housing (*n *= 1,150) plus a small number living off campus (*n *= 18).

*Alcohol consumption in high school and college *was measured as the typical number of drinks consumed per day, on days in which the student drank alcohol during the past 12 months. Rather than a categorical measure, we elected to use this continuous measure in order to represent the variability that occurs within the higher levels of alcohol consumption, which is sometimes obscured with categorical measures (e.g., 5 or more drinks per day) [38]. This consideration is especially relevant for studying college students, a population for which very high levels of drinking is not uncommon. The computerized screening questionnaire that was administered to obtain data on high school drinking levels allowed for a series of 11 integer options using "radio button" responses, ranging from one drink per day to "eleven or more", rather than allowing students to enter a continuous number. Because only a small number of students (2.1%) chose "eleven or more" drinks per day, we set their drinks/day to 11 to derive a continuous measure. Because the second assessment was a personal interview administered during the first year of college, the number of drinks consumed on a typical drinking day was captured as an open-ended, continuous variable, where the highest response obtained was 20. For both high school and college, alcohol consumption was coded as zero for students who did not drink at all in the past year. Sample means were 4.0 (*SD *= 2.8) and 4.5 (2.9) for high school and college, respectively. We found this measure to demonstrate a high degree of concordance with more detailed measures of college drinking, including calendar data using the Timeline Followback method.

*Parental monitoring during the last year of high school *was measured with a slightly adapted version of the parental monitoring scale, which was developed by Capaldi and Patterson [39] and later used by Chilcoat et al. [40]. This nine-item scale includes questions on the child's perception of parental rule-setting, supervision, consequences and monitoring; each item is scored on a five-point scale. Our adaptations involved minor word changes that made the scale more appropriate for older adolescents. The version used in this study had good psychometric properties (Cronbach's α = 0.76). Actual scale scores ranged from 9 to 45 points (*M *= 29.2; *SD *= 6.3), with a higher score representing a higher level of parental monitoring. Table 1 displays the nine scale items with their corresponding sample means and standard deviations, as well as the scale mean and standard deviation.

**Table 1:** The Parental Monitoring Scale administered during the summer prior to entry into college (*n *= 1,253).

*Thinking back over your last year in high school...*^a^	**Mean**	***SD***
1. When you got home from school, how often was an adult there within an hour of you getting home?	3.4	1.2
2. When you went to parties, how often was a supervising adult present at the party?	2.4	1.0
3. When you wanted to go to a party, how often did your parents confirm that an adult would supervise the party?	2.4	1.3
4. How often would your parents know if you came home an hour or more late on weekends?	3.7	1.3
5. When you broke a rule set by your parents, for example, coming home past curfew, did your parents take away privileges?	2.9	1.3
6. How often before you went out would you tell your parents when you would be back?	3.8	1.1
7. When your parents were not home, how often would you leave a note for them about where you were going?	3.6	1.3
8. When you went out and your plans unexpectedly changed, how often did you call your parents to let them know?	3.2	1.2
9. When you went out, how often did you let your parents know where you planned to go?	3.7	1.0

**TOTAL PARENTAL MONITORING SCALE SCORE**	**29.2**	**6.3**

^a ^Response Categories: 5 = All of time; 4 = Most times; 3 = Sometimes; 2 = Hardly ever; 1 = Never

### Statistical analyses

Consistent with literature cited above, we expected that parental monitoring would have a protective effect on high school drinking and that high school drinking would correlate strongly with college drinking. We further hypothesized that parental monitoring would have an indirect protective effect on college drinking via its influence on high school drinking. To test these hypotheses, we performed a series of ordinary least squares multiple regressions predicting (1) high school drinking on the basis of parental monitoring, (2) college drinking on the basis of parental monitoring, and (3) college drinking on the basis of both parental monitoring and high school drinking. For each analysis, hypothesized effects were first evaluated at the bivariate level as were the effects of several control variables (race, sex, mother's education, religiosity, current living situation, time in college, and combined SAT score). Next, all explanatory variables were evaluated together in a series of multiple regression models. In the final model, the first-order interaction term of parental monitoring with high school drinking was included to test for a possible moderating effect. Because students were interviewed at various times during their first year in college, the combined models included time in college (i.e., the number of months from arrival on campus to the interview date) to adjust for any potential confounding effects related to the timing of the interview. Effect size for each explanatory variable was evaluated using the semipartial *r*^2 ^(*sr*^2^) statistic, which represents the proportion of variance in the outcome variable uniquely explained by an explanatory variable, while holding constant the remaining explanatory variables in the model [41].

## Results

### Sample characteristics

As can be seen in Table 2, males and females were almost equally represented in the sample. Students were on average 18.2 years old, and 71% were White. Almost three-quarters had mothers with a college degree or more, and the mean SAT score was 1269. Half the sample indicated that religion was moderately or extremely important in their lives.

**Table 2:** Sample characteristics (*N *= 1,253)

**Sex**	
% Male	48.6
% Female	51.4
**Age **(Mean, *SD*)	18.21 (0.51)
**Race**	
% White	71.0
% Non-White	29.0
**Mother's Education**	
% Less than high school	1.3
% High School or GED	15.2
% Some college or technical school	10.1
% Bachelor's degree	38.0
% Graduate school	35.5
**SAT Score **(Mean, *SD*)	1268.16 (119.3)
**Importance of Religion**	
% Not Important	25.8
% Slightly Important	24.2
% Moderately Important	31.0
% Extremely Important	19.0

### Predictors of high school alcohol consumption

Table 3 shows the results of the regression models predicting high school drinking. As can be seen from the bivariate results, sex, race and religiosity were all significantly related to high school drinking (*p *< .001), with males, White students, and students with lower religiosity having higher levels of alcohol consumption in high school. As expected, parental monitoring was strongly negatively related to high school drinking, such that number of drinks per day decreased as the parental monitoring score increased (*b *= -0.13, *t*(*df*) = -10.49(1,194), *sr*^2 ^= .08, *p *< .0001). Interestingly, the results of the multivariate model indicate that while sex and race had significant independent effects on high school drinking level, the effect of religiosity become non-significant once sex, race and parental monitoring were held constant (*p *= .56). Additional post-hoc analyses suggested that the effect of religiosity in the model was reduced mainly by the addition of parental monitoring, rather than sex or race. The magnitude of the parental monitoring effect remained essentially unchanged by the addition of these covariates (*b *= -0.12, *t*(*df*) = -9.26(1,092), *sr*^2 ^= .07, *p *< .0001.

**Table 3:** Results of linear regression predicting alcohol consumption in high school ^a ^(*n *= 1,100).

	**Bivariate Models**						**Multivariate Model^c^**				
	***b***	***SE***	***t*(*df*)**	***sr***^2^	***p***		***b***	***SE***	***t*(*df*)**	***sr***^2^	***p***
**Parental Monitoring Score**	-.13	.01	-10.49 (1,194)	.08	<.0001		-.12	.01	-9.26 (1,092)	.07	<.0001
**Sex **[Reference = Female]	.97	.16	6.14 (1,249)	.03	<.0001		.69	.16	4.32 (1,092)	.01	<.0001
**Race **[Reference = Non-White]	1.37	.17	7.91 (1,246)	.05	<.0001		1.26	.18	7.02 (1,092)	.04	<.0001
**Religiosity**^b ^[Reference = Slightly/Not Important]	-.56	.16	-3.46 (1,243)	.01	.0006		-.10	.16	-.59 (1,092)	<.01	.56
***R***^2^							**.14**				
***F (df, df) p***							24.82 (7, 1,092) *p *< .0001				

Effects were evaluated using the null hypothesis test of *b* = 0 (tested as: *b/SE*) which evaluates the unique contribution of a variable in a regression equation.

^a ^High school alcohol consumption was defined as the typical number of drinks per drinking day during the past year at the screener.

^b ^Religiosity was dichotomized into a binary variable (i.e., extremely/moderately vs. slightly/not).

^c ^As a proxy for socioeconomic status, the effect of mother's education was held constant in the multivariate model. Effect size (*sr*^2^) for each explanatory variable was as follows: parental monitoring score (.07), sex (.01), race (.04), religiosity (<.01), mother's education (<.01).

### Predictors of college alcohol consumption

Table 4 presents the results of the regression analyses predicting college drinking, as measured by the number of drinks/drinking day. Without controlling for other covariates, parental monitoring during high school exerted a protective effect on college drinking level (*b *= -0.11, *t*(*df*) = -8.26(1,189), *sr*^2 ^= .05, *p *< .0001), albeit less strongly than the effect on high school drinking level (shown in Table 3). Sex, race, SAT score, religiosity and living with parents were also significantly related to college drinking, similar to high school drinking. Additionally, SAT score was positively associated with college drinking (*b *= 0.29, *t*(*df*) = 4.27(1,233), *sr*^2 ^= .01, *p *< .0001), whereas living with parents was negatively associated (*b *= -2.50, *t*(*df*) = -7.82(1,245), *sr*^2 ^= .05, *p *< .0001) Time in college, which was included as a control variable, did not contribute significantly to college drinking (*p *= .42).

**Table 4:** Results of linear regression models predicting alcohol consumption in college ^a   ^among 1,086 first-year college students ^c^.

	**Bivariate Models**						**Model 1**						**Model 2**						**Model 3**				
	***b***	***SE***	***t*(*df*)**	*sr*^2^	***p***		***b***	***SE***	***t*(*df*)**	*sr*^2^	***p***		***b***	***SE***	***t*(*df*)**	*sr*^2^	***p***		***b***	***SE***	***t*(*df*)**	*sr*^2^	***p***

**Parental Monitoring**	-.11	.01	-8.26 (1,189)	.05	<.0001		-.08	.01	-6.26 (1,075)	.03	<.0001		-.01	.02	-.52 (1,073)	<.01	.60		-.02	.01	-1.43 (1,074)	<.01	0.15
**High School Drinking**^a^	.66	.02	30.05 (1,243)	.42	<.0001								.63	.10	6.01 (1,073)	.02	<.0001		.58	.02	23.52 (1,074)	.27	<.0001
**Interaction **[PM * HS Drinking]													.00	.00	-.50 (1,073)	<.01	.62					
**Sex **[Reference= Female]	1.69	.16	10.74 (1,245)	.08	<.0001		1.62	.16	10.03 (1,075)	.07	<.0001		1.11	.13	8.33 (1,073)	.03	<.0001		1.10	.13	8.32 (1,074)	.03	<.0001
**Race **[Reference= Non-White]	1.69	.18	9.62 (1,243)	.07	<.0001		1.45	.18	7.96 (1,075)	.05	<.0001		.72	.15	4.75 (1,073)	.01	<.0001		.72	.15	4.76 (1,074)	.01	<.0001
**Religiosity**^b ^[Reference= Slightly/Not Important]	-.53	.16	-3.22 (1,238)	.01	.001		-.03	.16	-.19 (1,075)	<.01	.85		.04	.13	.31 (1,073)	<.01	.76		.04	.13	.32 (1,074)	<.01	0.75
**Living with Parents/Relatives**	-2.50	.32	-7.82 (1,245)	.05	<.0001		-2.21	.33	-6.63 (1,075)	.03	<.0001		-1.32	.27	-4.81 (1,073)	.01	<.0001		-1.32	.27	-4.81 (1,074)	.01	<.0001
**Time in college **(months)	-.03	.04	-.81 (1,245)	<.01	.42		-.05	.03	-1.49 (1,075)	<.01	.14		.03	.03	.98 (1,073)	<.01	.33		.03	.03	1.00 (1,074)	<.01	0.32
**Combined SAT/100**	.29	.07	4.27 (1,233)	.01	<.0001		-.12	.07	-1.60 (1,075)	<.01	.11		.04	.06	.59 (1,073)	<.01	.55		.04	.06	.60 (1,074)	<.01	0.55
***R***^2^							.22						.48						.48				
***F (df, df) p***							30.02 (10, 1,075) *p *< .0001						83.92 (12, 1,073) *p *< .0001						91.59 (11, 1,074) *p *< .0001				

Effects were evaluated using the null hypothesis test of *b *= 0 (tested as: *b/SE*) which evaluates the unique contribution of a variable in a regression equation.

^a ^High school and college drinking were defined as the number of drinks per drinking day during the past year.

^b ^Religiosity was dichotomized into a binary variable (i.e., extremely/moderately vs. slightly/not important).

^c ^As a proxy for socioeconomic status, the effect of mother's education was held constant in the multivariate models.

In the first multivariate model that controlled for all the covariates but did not control for high school drinking (see Model 1), the model estimates were essentially unchanged with two important exceptions. First, religiosity did not retain significance once parental monitoring and other covariates were held constant. Second, the effect of SAT score on college drinking was reduced quite substantially and became non-significant. In the second multivariate model (Model 2) where high school drinking was included, high school drinking was strongly related to college drinking (*b *= 0.63, *t*(*df*) = 6.01(1,073), *sr*^2 ^=.02, *p *< .0001), whereas the effect of parental monitoring became non-significant (*p *= .60). The interaction between parental monitoring and high school drinking did not contribute significantly to the model (*p *= .62). Model estimates for race and sex were reduced appreciably from Model 1, but retained statistical significance. Finally, Model 3 excludes the interaction between parental monitoring and high school alcohol consumption, and shows that the model explains 48% of the variance in college drinking. Moreover, the effect of high school drinking accounted for a large proportion of variance in college drinking (*sr*^2 ^= .27) whereas parental monitoring remained non-significant (*sr*^2 ^< .01). In summary, we directly tested our mediation hypothesis in the models shown in Tables 3 and 4, and found that parental monitoring predicts both high school and college drinking, but that the addition of high school drinking to the college drinking model obscures the effect of parental monitoring. Thus, results are consistent with a mediational model, such that the effect of parental monitoring on college drinking is mediated by, rather than moderated by, high school drinking level, holding constant the effects of sex, race, time in college, SAT score, and mother's educational level. Importantly, the magnitude of the model estimates did not change appreciably when sampling weights were applied to correct for any potential impact of oversampling for experienced drug users (data not shown).

## Discussion

Consistent with prior studies with younger adolescents [32], this study observed that higher levels of parental monitoring and supervision were associated with lower levels of high school alcohol consumption, independent of sex, race and religiosity. Moreover, it appears that parental monitoring may exert an indirect protective effect on college drinking through its effect on high school drinking.

The findings of this study must be tempered by several limitations. First, because students were sampled from one university, findings may not be generalizable to other college student populations, for example at smaller colleges or where different demographic characteristics or geographic regions are represented. Second, this study only measured one facet of college drinking, future studies should attempt to see if other measures of drinking provide varying results. The distribution of responses for our drinking measure was skewed positively for both high school drinking (.35) and college drinking (.78), because the proportion of non-drinkers was higher than expected in a normal distribution (15.8% and 7.7%, respectively). However, we obtained similar results from replicating the multivariate models in a restricted sample that excluded non-drinkers. Nevertheless, results should be interpreted cautiously whenever the criterion variable in a regression model is not normally distributed. Third, although a standard measure of parental monitoring was used in this study, the construct being measured might be highly correlated with positive child characteristics and not simply parent behaviors. For example, the item pertaining to leaving a note is both a reflection of the child's willingness to conform to prosocial behavior and perhaps the expectation set by parents about the need to leave a note. Future studies should attempt to disentangle the relative effects that are more child-driven from behaviors that are under the parents' control, such as taking away privileges if a student comes home past curfew. Fourth, it is important to recognize that the measure of parental monitoring used in the present study is limited to only one facet of effective parenting, namely the student's perception of parental monitoring, and may not necessarily reflect actual parent behavior. Moreover, our measure may be a proxy for the presence of other parenting behaviors, such as parental disapproval of underage drinking [42], and effective parent-child bonding and communication [43,44], which also have been found to be associated with risk of adolescent tobacco and alcohol use.

Fifth, the observed protective effect of parental monitoring during the last year of high school could be interpreted to be a marker of effective parenting throughout adolescence. Unfortunately, the current study did not measure parent monitoring and supervision during earlier developmental periods.

This study is also limited in its ability to explain the mechanism by which parental monitoring exerts its protective effects. Several studies have suggested that parental monitoring might limit affiliation with deviant peers [45-47], or might be a sign of lower family conflict, higher quality and/or quantity of communication, greater parental warmth, or greater parent-child attachment [27,36,48,49]. Also, higher levels of willingness to cooperate with parents might indicate a desire to model healthy drinking behaviors of parents, which may be subsequently translated into a reduced risk for heavy drinking in college [36]. Prior evidence indicates that children develop positive attitudes about alcohol use when their parents drink more and hold positive alcohol-related expectancies [1]. Conversely, adolescents whose parents have negative attitudes toward alcohol and disapprove of underage drinking, show lower levels of alcohol use, are more likely to engage with peers who also do not drink, and have a higher level of self-efficacy for alcohol refusal [30]. This study did not measure the possible direct influence of peer alcohol use, or individual expectancies related to alcohol use, which have been shown to be important predictors of college alcohol consumption [50]. Future research should aim to understand the interplay between these sorts of family characteristics and college alcohol drinking patterns, and examine how a child's temperament characteristics may influence this already complex chain of variables. In addition, other environmental characteristics, such as price [51] or campus policies, should be examined.

## Conclusion

Despite the above limitations, the present findings have implications for both prevention and future research. Based on our findings, we speculate that with regard to prevention, the results extend support for parental monitoring and supervision during the high school years as a strategy to reduce adolescent drinking. An interesting policy debate exists around the degree to which parents should be involved in monitoring their college student's behavior related to alcohol consumption, since, legally their children are adults. There is more, but not unanimous, agreement pertaining to involving parents when there is a life-threatening situation involving their child. For example, not all universities have mandatory parental notification policies in cases of alcohol poisoning. The issue raised by the results of this study is whether it might be useful to engage parents at an earlier stage – namely, in prevention strategies to reduce underage drinking during college. While the results of this study showed that parental monitoring during the last year of high school was associated with reduced levels of high school drinking, and high school drinking levels in turn predicted college drinking levels, it would be important to know whether continued parental involvement in college had additional benefits on reductions in college student drinking over and above the effects of high school drinking levels. Evaluations of programs that focus on modifying parenting practices have shown promising results in reducing risk among younger adolescents. Providing consistent discipline, setting rules, monitoring adolescents' activities, providing positive reinforcement, and communicating with adolescents are all parenting tools that have proven efficacious in reducing and delaying adolescent drinking and risk-taking behaviors [25,26,31-33,52-54]. Two programs that have been particularly effective in this regard are Preparing for the Drug-Free Years and the Iowa Strengthening Families Program, both of which focus on competency training sessions for parents and include adolescents in part of the trainings [55].

To our knowledge, few interventions have focused specifically on parental engagement strategies to reduce college drinking [28]. In their recent report "Wasting the Best and the Brightest: Substance Abuse at America's Colleges and Universities", the National Center for Addiction and Substance Abuse at Columbia University (CASA) strongly advocates for parental involvement as part of a comprehensive strategy to reduce underage drinking on campus.

There are a variety of possible ways to involve parents in prevention. First, at the very least, parents can communicate with their college-age child about campus policies related to underage drinking and illicit drug use. For this to happen, campus officials must inform parents of these policies, at orientation and on an ongoing basis during the time their child is in college. CASA notes that close to 90% of colleges report that their policies are available for inspection by parents, either through direct communication, or through the college website [56].

Second, parents could be encouraged by colleges to express disapproval of underage drinking while their child attends college. Research shows that college students who report that their parents have permissive attitudes about underage drinking and substance use are more likely to engage in these behaviors [57]. This is contrary to the belief that college binge drinkers are the ones who were prohibited drink in high school. In a blog on the Wall Street Journal website that was focused on an article about college parental notification guidelines of alcohol and drug use, comments such as this were posted,

"The only reason for college binge drinking is prohibition. Kids that binge drink in college are the same ones that were raised by 'responsible' parents who did not let their kids try a drop of alchohol (sic) at home.

Once kids get to college and away from parents' relatively frequent control they go on a rampage. Binge drinking can easily be resolved in late teenagehood by educating kids how to drink, what it means to drink a lot, and what hangover is [58]."

However, our study has shown that binge drinking in high school predicts college binge drinking. In addition, the CASA survey found that 70% of college students reported that their parents' concerns or expectations either somewhat or very much influenced whether or how much they drank, smoked, or used other drugs, and that parental attitudes were significantly related to the likelihood to binge drink, use marijuana, and smoke tobacco.

Third, colleges could encourage parents to play an important role in recognizing early warning signs of alcohol abuse and intervening by facilitating access to services. If parental notification policies were in place when a student receives a citation from residence life for underage drinking, then parents could encourage their student to undergo a comprehensive assessment, which includes exploration of underlying mental health issues. The 1998 amendments to Part E, Section 952 of the Higher Education Act of 1965 (PL 105–244) allow institutions of higher education to notify parents of students who are under the age of 21 when the student has committed a school disciplinary violation involving alcohol or a controlled substance. The United States Department of Education clarified the ruling in 2000 by stating that "school officials may notify parents whenever they determine that a disciplinary violation has occurred, and that those determinations can be made without conducting a formal disciplinary preceding or hearing [59]." The point to be made is that the parental notification does not necessarily imply a punitive action; rather the policy can serve a critical purpose to guide the student toward services that may be necessary to avert further problems downstream [60].

Although many students may be physically separated from their parents once they begin college, future studies are needed to investigate whether or not certain parental practices with college-age children (e.g., frequent appropriate communication, parental support and encouragement, and monitoring of peer group activities) might be helpful in reducing the risk of problematic alcohol consumption. In the 2002 landmark report "A Call to Action: Changing the culture of drinking at US colleges," several potential strategies for reducing excessive drinking, such as social marketing campaigns and environmental strategies were described. Evaluation research should continue to understand the extent to which these types of prevention strategies are effective. This report offered scant information about the protective role of parents except to say that parents should be aware of campus policies on alcohol and illicit drugs [61]. Arguably, traditional forms of parent-child interaction such as parent monitoring and supervision will naturally decrease as adolescents get older and leave home for college. An important aim for future research should be to define the characteristics of an appropriate parent-child relationship that is associated with low risk for the development of alcohol and other drug problems. Defining this balance between parental guidance and youth autonomy is challenging since too much monitoring in the college years might cause strain in the parent-student relationship and increase the risk for negative outcomes. Research in this area will depend on the development of instruments that can measure changes in the parent-child relationship during the transition from older adolescence through young adulthood.

In summary, the transition to college marks a high-risk period for escalation of alcohol consumption. Parents and prevention practitioners can benefit from evidence that points to specific parenting practices that might help reduce the risk for heavy drinking, while at the same time allow for appropriate levels of autonomy that are critical for young adult development. Colleges should invest in initiatives that involve parents as partners in communicating the message to students about the risks of heavy drinking and promote appropriate levels of parental monitoring through the college years.

## Abbreviations

CASA: Center for Addiction and Substance Abuse; PL: Public Law; *M*: Mean; *SD*: Standard Deviation

## Competing interests

The author(s) declare that they have no competing interests.

## Authors' contributions

AA, KO'G, and EW contributed to the overall scientific direction of the project. KC and KV managed the day-to-day operational aspects of data collection. KV supervised staff involved in data collection. VK, KC and KO'G performed the statistical analyses. AA, VK, KO'G, KC, KV, and EW assisted with writing. All authors read and approved the final manuscript.
